# Associations between nature exposure, screen use, and parent–child relations: a scoping review protocol

**DOI:** 10.1186/s13643-023-02367-2

**Published:** 2023-11-16

**Authors:** Marina Torjinski, Sharon Horwood

**Affiliations:** 1 School of Psychology, Deakin University, Locked Bag 20000, Geelong, 3220 Australia; 2 ARC Centre of Excellence for the Digital Child, Education Building (E Block), Level 4, QUT Kelvin Grove campus, Brisbane, Australia

**Keywords:** Scoping review protocol, Screen-use, Parent–child relations, Green space, Nature therapy, Children

## Abstract

**Background:**

Today’s youth are growing up in an evolving digital world, and concerns about the potential detrimental effects of excessive screen use on biopsychosocial outcomes in childhood are mounting. Parents worry about the impacts of screen-use on their children’s wellbeing but at the same time frequently fail to meet their own ideal screen time limits regarding their children’s screen use. There is an opportunity to shift research focus away from inflexible and often unrealistic childhood screen time guidelines towards exploration of positive parenting strategies that may have multiple beneficial and significant effects on children’s screen-related outcomes. An emerging body of literature suggests that screen time and nature exposure act on psychosocial outcomes in contrasting ways. There is evidence to suggest that exposure to natural environments may counteract some of the potential negative psychosocial effects of excessive screen use; however, this relationship is poorly understood. The overarching aim of this scoping review is to source, categorise, and synthesise existing research exploring the associations between nature exposure, screen use, and parenting across childhood.

**Methods:**

This mixed-methods systematic scoping review will be conducted following Arksey and O’Malley’s framework with methodological enhancements from Levac and associates and recommendations from the Joanna Briggs Institute’s methodological guidance for conducting scoping reviews. Five electronic databases will be searched from August 2022 onwards. Two reviewers will independently screen titles, abstracts, and full-text articles. Peer reviewed articles related to the constructs of nature exposure, screen use, and parent/child relations will be considered in the context of early to late childhood. Study characteristics will be collated using a data charting tool collaboratively developed by the research team. Evidence will be presented using tabular and textual form and described using qualitative thematic analysis.

**Discussion:**

This review will gather information about how key definitions are conceptualised, defined, and measured across the literature and map existing trends and areas for future research. It is intended that this review will inform and guide future research direction, recommendations, and programs aimed at supporting parents to navigate the challenges of parenting in a digital age.

**OSF registration:**

https://doi.org/10.17605/OSF.IO/TFZDV

**Supplementary Information:**

The online version contains supplementary material available at 10.1186/s13643-023-02367-2.

Modern family life is becoming increasingly characterised by an uptake of new, portable, and connected technology by children of increasingly younger ages. A rapidly growing body of research suggests that excessive screen use can have detrimental effects on physical, psychological, and social outcomes in early to late childhood [1–3]. National screen time guidelines recommend that parents limit their children’s exposure to screen devices [4]; however, novel findings reveal that parents struggle to uphold their ideal screen time limits despite knowledge of the harms and intention to reduce their child’s screen use [5]. As described in Bronfenbrenner’s ecological systems theory [6], early to late childhood is characterised by sensitive developmental stages shaped by interdependent and bidirectional interactions between the parent and child. Accordingly, screen-related outcomes in childhood cannot be observed in isolation from parent/child dynamics, parenting beliefs, practices, and other parent factors. Research exploring parental perceptions of childhood screen use has identified a reciprocal relationship whereby children’s screen-related behavioural difficulties interact with perceived parental stress—in turn challenging parental capabilities to effectively manage their child’s difficult behaviours [1]. However, the mechanisms and impacts of this parent–child interaction are poorly understood.

Given that digital awareness campaigns may not translate into effective screen-limiting practices within the home, it may be necessary to seek a more practicable approach to supporting parents to mitigate the potential harms of childhood screen use. In the family context, parental self-efficacy can be conceptualised as a parent’s beliefs and attitudes about their ability to parent effectively and is significantly related to parenting behaviours and practices [7, 8]. An emerging body of literature suggests that parents who have higher parental self-efficacy are more likely to engage in parenting practices that impose greater screen time restrictions on childhood screen use [9]. In addition, parental self-efficacy is an important variable to target when developing strategies to encourage healthy childhood screen use. One challenge to parental self-efficacy is the semantic nature of childhood screen time guidelines. Typically, childhood screen time guidelines focus on what parents should not do. For example, parents should not let their children use screens for more than 2 h per day. Negatively framed screen-related messaging can often evoke feelings of parental guilt that may corrode parental self-efficacy beliefs [5]. Positively framed messaging may be a more effective determinant of behaviour change than messaging that evokes negative emotions [10]. In a digitally evolving world, where exposure to screen devices in inevitable, there is a need to shift away from messages focused solely on screen time and focus on encouraging both positive screen uses and positive parenting interventions that may have direct and indirect impacts on screen-related child behaviours.

Despite mixed findings, exposure to natural environments or ‘green time’ is a growing area of scientific inquiry, and research suggests that exposure to natural spaces may promote psychological [11, 12], cognitive [13], and social [14–16] wellbeing. Globally, an increase in childhood screen use has also corresponded with reductions in time children spend in natural environments, and the positive psycho-social benefits of nature exposure appear to map inversely with screen-related child health outcomes [17]. A very limited body of evidence has suggested that exposure to natural spaces has the potential to ameliorate the negative effects of screen exposure in childhood; however, the reciprocal effects and mechanisms underpinning this relationship are poorly understood [17]. To the best of our knowledge, one previous scoping review has collated evidence exploring the effects of ‘green time’ and ‘screen time’ on psychological outcomes in childhood and adolescence [17]. Although the literature was highly heterogenous, results of cross-sectional studies consistently revealed that higher screen time was associated with adverse psychological outcomes, whilst higher green time was associated with positive psychological outcomes. Since the review, additional studies of relevance have come to light however there is a paucity of research exploring the ways in which nature exposure and screen use interact to influence parent–child outcomes and relational wellbeing. This is a significant research gap as the reciprocal nature of parent–child interactions underpins a broad spectrum of child outcomes. Research suggests that parental attitudes about play environments significantly influence children’s play and activity preferences [18]; therefore, parent-variables influencing family routines and dynamics need to be further explored in the context of childhood screen use and outdoor play. In seeking to theoretically frame the interplay of nature exposure and childhood screen use within the context of parent–child relations, this review will draw on two developmentally relevant contemporary relational frameworks (interactional theory of childhood problematic media use [19] and Izenstark and Ebata’s integrative model of family-based nature activities [20]). A greater understanding of the available evidence will further the field of study by informing and guiding future research direction, clarifying key concepts and shedding light on novel ways to support parents to navigate the challenges of parenting in a digital age.

Preliminary searches were conducted in PsycINFO (EBSCO) using key variable terms in varying combinations to (a) identify the most suitable review type and determine feasibility of the proposed review and (b) search for keywords, terms, and phrases related to the three variables of interest to formulate a detailed and accurate search syntax. For the purpose of this review, screen use will be conceptualised as use of any technological device including televisions, computers, and modern touch-screen devices such as tablets or smartphones. Nature exposure will be conceptualised as any human contact with the natural environment in an outdoor setting; this may include green spaces such as forests, blue spaces such as beachscapes, or urban outdoor spaces such as tree-lined streets and parks.

A priori searches identified that the relevant literature was characteristic of highly heterogenous population samples, variable definitions, study designs, and methodologies. There appears to be a lack of studies exploring all key concepts in combination, necessitating the need to search literature according to three themes: nature exposure and screen use, nature exposure and parent–child relations, and all three concepts together. Consequently, a systematic mixed method scoping review was decided as the most appropriate review type. Inclusion of a wide range of study types and methodologies will allow for a more comprehensive synthesis of key concepts. Furthermore, as the review will explore parent–child dynamics, inclusion of qualitative studies is necessary to capture findings investigating parental perceptions, attitudes, and processes with respect to childhood screen use and nature exposure.

## Study objectives

The review objectives are as follows:

- To map the scope of existing literature exploring nature exposure, screen use and parent–child relations across childhood
- To gather information about how key definitions for screen use, nature exposure and parent–child relations are conceptualised, defined, and measured across the literature
- To synthesise findings from a range of literature and identify main study findings, existing gaps, limitations, and recommendations

## Methods

This mixed-methods systematic scoping review will be conducted following Arksey and O’Malley’s framework [21] with methodological enhancements from Levac and associates [22] and further recommendations from the Joanna Briggs Institute’s methodological guidance for conducting scoping reviews [23]. This framework proposes five sequential stages to developing a scoping review: (a) identifying the research question, (b) identifying relevant literature, (c) selecting studies, (d) mapping the data, and (e) summarising, synthesising, and reporting the results. This protocol is registered with the Open Science Framework (registration ID: https://doi.org/10.17605/OSF.IO/TFZDV) and has followed reporting recommendations from the preferred reporting items for systematic review and meta-analysis protocols [23] (see Additional file 2). The Preferred Reporting for Systematic Reviews Checklist [24] will be adhered to when final review output is reported. All data will be saved on a password protected secure Deakin University server.

### Step 1: Identifying the research question

The review will be guided by the following research question: *What is the scope of existing literature, including construct definitions, major findings, limitations, and areas for future research, that explores nature exposure, screen use and parent*–*child relations across childhood?* For the purpose of this review, ‘nature exposure’ will be conceptualised as exposure to any outdoor space that is characterised by elements of the natural environment (see Table 1 for key definitions). This definition precludes time outdoors where features of natural environments are not specified (e.g. outdoor basketball court).

**Table 1:** Population-concept-context framework

Population	*Children:* Children aged 0–12, both typically and non-typically developing *Parents and caregivers*: A biological or non-biological primary legal carer to a child between 0 and 12 years, who lives with the child full-time (i.e. more than 5 days a week)
Concept	*Nature exposure:* Exposure to any outdoor space that is characterised by elements of the natural environment (greenery, waterways, mountains, etc.) *Screen use:* Relates to use of any technological device, including traditional modes such as television and computers, as well as modern touch-screen devices such as smartphones and tablets
Context	Global Thematic context (education, medicine, health-sciences, psychology) Last 10 years

### Step 2: Identifying relevant literature

The Population-Concept-Context framework (see Table 1) was used to guide the conceptualisation of key review questions, eligibility criteria for study inclusion, and the study selection process [23]. Preliminary searches were conducted in PsycINFO (EBSCO) using key variable terms in varying combinations to develop a targeted scope for eligibility criteria and search terms. Article subject headings and titles were scanned for alternative/additional definitions for key concepts. Where search results generated voluminous or non-specific articles, specific search terms were removed from the search strategy. For example, freestanding search terms such as ‘technology’ and ‘nature’ were either removed altogether or adjoined with other terms using Boolean operators for a more targeted search output. Inclusion criteria will be (a) peer reviewed studies published in English from 2012 onwards with either quantitative, qualitative, or mixed-methods design and (b) studies whose participants are children aged 0–12 years and/or primary caregivers (parents or carers) to children aged 0–12 years. A range of study methodologies (including randomised control trial, cross-sectional, and longitudinal) will be considered due to the explorative nature of the current review, novelty of the conceptual field, and heterogeneity of study designs. Exclusion criteria will be a) reviews b) unpublished data and c) grey literature. The screening and selection process will be represented by the Preferred Reporting Items for Systematic Reviews and Meta-analyses Flowchart Extension for Scoping Reviews [24]. Prior to implementation, the search strategy quality will be independently reviewed by two liaison-librarians, using The Peer Review of Electronic Search Strategies Evidence-Based Checklist [25]. The review search will be run through five electronic interdisciplinary and discipline-specific databases covering the conceptually related fields of health and medicine, psychology, and education. The following databases will be searched (from August 2022 onwards): PsycINFO (EBSCO), MEDLINE complete (EBSCO), ERIC (EBSCO), EMBASE and the Cochrane library. A structured step-by-step search strategy using keywords and subject terms will be used for each database (an additional file shows this in more detail (see Additional file 1)). Boolean operators and truncations will be used in various combinations for search parsimony. Only articles related to the constructs of nature exposure and screen-use and/or parenting will be considered in the context of early to late childhood (up to 12 years old). Additional studies will be obtained through snowballing of all publications identified for inclusion. The retrieved articles will be collated and managed through Covidence, and duplicates will be removed.

### Step 3: Selecting studies

Two reviewers will independently screen titles, abstracts, and selected full-text articles. Screening of abstracts will be an iterative process; study selection will be discussed by the review team at the start, middle, and end of the abstract review process, and refinements to the inclusion/exclusion criteria will be made if needed [22]. Discrepancies between reviewer decisions will be handled by a third reviewer. Articles that do not meet eligibility criteria will be removed and exclusion reasons will be documented along with the document source. Due to the nature and purpose of this scoping review, a formal risk of bias assessment was not deemed necessary [21].

### Step 4: Mapping the data

A customised data charting form (see Table 2) has been adapted from the template data extraction instrument for scoping reviews in the Joanna Briggs Institute Manual for Evidence Synthesis [23]. This charting tool will be collaboratively developed through an iterative process by the research team to determine which data needs to be extracted from articles to answer the research questions and will be updated throughout the extraction process. Following recommendations from Levac and associates [22], the research team will meet following data extraction for five studies, to establish whether the extraction process is capturing information aligned with the review aims and research questions.

**Table 2:** Initial data extraction charting template

Category	Description
Article data	
Title	Full title of the article
Author(s)	Who is/are the author(s)?
Publication year	When was the study published?
Country	In which country was the study conducted?
Study characteristics	
Key concepts	Does the study focus on nature exposure, screen use, parent/child relations or a combination of these?
Aims/purpose	What were the study aims and objectives?
Concept/variable definitions	How were key concepts/variables defined?
Population characteristics	
Sample size	How many people participated in the study?
Participant role	Parents, mothers, fathers or carers?
Age	Participants’ age
Gender	Participants’ gender
Setting	For example, home, school, recreation, etc
Methodology	
Measures	For example, named measure vs made up items
Data collection methods	For example, surveys, interviews, observational
Analysis type	How were findings analysed?
Confounding variables	For example, exercise
Knowledge contribution	
Key findings	What were the main study results?
Key limitations	What were the main study limitations?
Key recommendations	What were the main recommendations for future research?

### Step 5: Summarising, synthesising, and reporting the results

Evidence will be collated and described following Arksey and O’Malley’s methods [21], with recommendations from Levac and associates [22]. First, a numerical summary of information obtained from the data extraction charting template will be provided in tabular form, describing key characteristics of included studies (e.g. study design, intervention, population and outcome measures). A textual summary of the data will be analysed and described using qualitative thematic analysis. Information within the populated data charting tool will be hand coded and then further developed by identification of overarching themes. Relationships and connections between themes will be mapped using mind-mapping software and critically discussed by all authors throughout an iterative mapping process. Discussion of results will reflect the final thematic categories. Results will be reported in alignment with the review objectives and research questions and interpreted in view of future research direction and implications on recommendations, policies, and practices within the conceptual field.

## Discussion

The results of this scoping review will shed light on current developments in the field by providing an overview of the literature and identifying conceptual and methodological gaps and limitations. This review may inform future research direction and lead to the development of new recommendations and parenting interventions around childhood screen use. The broad nature of this review may help determine areas where more specific research questions need to be explored to progress the field (for example, developing more reliable and consistent ways to measure and define key constructs). This review may help tease out the unique role of nature exposure within broader areas of research, such as studies exploring outdoor health and movement behaviours as well as outdoor education and recreational therapies. The authors anticipate that this review will shed light on the ways in which screen use and nature exposure interact with family dynamics in the context of early to late childhood. The potential benefit of this area of research is that nature exposure may indirectly influence screen-related child outcomes by influencing parent/child wellbeing and strengthening the parent–child relationship. As a result of this review, it is anticipated that a rationale will be provided for why nature exposure should be further pursued through scientific enquiry as a potential parent–child intervention for improving screen-related child outcomes.

### Supplementary Information

**Additional file 1.** Associations between nature exposure, screen use and parent–child relations across childhood; example scoping review search strategy. This document illustrates the structured step-by-step search strategy string developed for each of the five electronic databases to be searched: PsycINFO (EBSCO), MEDLINE complete (EBSCO), ERIC (EBSCO), EMBASE and the Cochrane library. Keywords and subject terms have been adapted for each database.

**Additional file 2.** PRISMA-P Checklist- Nature exposure, Screen Use and Parent–child relations, a scoping review. This checklist has been adapted from Table 3 in Moher et al (2015): Preferred reporting items for systematic review and meta-analysis protocols (PRISMA-P) 2015 statement. *Systematic Reviews* 2015 4:1.

## Data Availability

Not applicable.
