# Supplementary material for: Associations between nature exposure, screen use, and parent–child relations: a scoping review protocol
Source: Syst Rev. 2023 Nov 16;12:217. doi: 10.1186/s13643-023-02367-2 (PMC10652600; doi:10.1186/s13643-023-02367-2)
Supplement: Supplementary file 1 — Additional file 1. Associations between nature exposure, screen use and parent–child relations across childhood; example scoping review search strategy. This document illustrates the structured step-by-step search strategy string developed for each of the five electronic databases to be searched: PsycINFO (EBSCO), MEDLINE complete (EBSCO), ERIC (EBSCO), EMBASE and the Cochrane library. Keywords and subject terms have been adapted for each database. [file 13643_2023_2367_MOESM1_ESM.docx]

### **Additional file 1: Associations between nature exposure, screen use and parent-child relations across childhood; example scoping review search strategy.**

**Search Strategy**

| **Database** | **Search strategy** | **Comments and alternative subject headings** |
| --- | --- | --- |
| ***APA Psycinfo (Via EBSCO****)* | S1  (TI(green OR nature OR forest OR outdoor OR wilderness) N3 (time OR space* OR exposure OR area* OR playground* OR environment* OR exercise* OR based OR play* OR school* OR experience* OR therap* OR bathing OR learning OR education OR immersion OR connectedness)) OR (AB(green OR nature OR forest OR outdoor OR wilderness) N3 (time OR space* OR exposure OR area* OR playground* OR environment* OR exercise* OR based OR play* OR school* OR experience* OR therap* OR bathing OR learning OR education OR immersion OR connectedness))  S2  TI greenspace* OR AB greenspace* OR TI “blue space*” OR AB “blue space*” OR TI “shinrin yoku” OR AB “shinrin yoku” OR TI “eco-therap*" OR AB “eco-therap*” OR TI greenness OR AB greenness  S3  DE "Wilderness Experience"  S4  S1 OR S2 OR S3  S5  (TI(screen OR screens OR “electronic device*” OR computer* OR hand-held OR media OR tablet* OR mobile-device* OR mobile-phone* OR television* OR I-pad* OR touch-pad* OR cell-phone* OR smart-phone* OR I-phone*) N3 (use OR exposure OR time OR behavio#r* OR addict*)) OR (AB(screen OR screens OR “electronic device*” OR computer* OR hand-held OR media OR tablet* OR mobile-device* OR mobile-phone* OR television* OR I-pad* OR touch-pad* OR cell-phone* OR smart-phone* OR I-phone*) N3 (use OR exposure OR time OR behavio#r* OR addict*))  S6  DE “digital gaming” OR DE “screen time” OR DE “smartphone use”  S7  S5 OR S6  S8  (TI(family OR families OR parent* OR mother* OR father* OR “parent-child” OR carer OR caregiver* OR guardian* OR mum* OR mom* OR dad* OR childrearing OR “child rearing” OR maternal OR paternal)) OR (AB(family OR families OR parent* OR mother* OR father* OR “parent-child” OR carer OR caregiver* OR guardian* OR mum* OR mom* OR dad* OR childrearing OR “child rearing” OR maternal OR paternal))  S9  DE "Family" OR DE "Family Relations" OR DE "Family and Parenting Measures" OR DE "Family Conflict" OR DE "Family Relations" OR DE "Child Discipline" OR DE "Childrearing Practices" OR DE "Parent Child Relations" OR DE "Parental Role" OR DE "Parent Child Communication" OR DE "Parental Attitudes" OR DE "Parent Report" OR DE "Parental Attitudes" OR DE "Parental Expectations" OR DE "Parental Characteristics" OR DE "Parental Role" OR DE "Parenting Style" OR DE "Parental Involvement" OR DE “family therapy” OR DE “family intervention” OR DE “mother-child relations” OR DE “father-child relations”  S10  S8 OR S9  S11  S4 AND S7 AND S10 | Limiters:  Peer reviewed.  2012-2022.  Human Subjects.  English. |
| ***MEDLINE complete (Via EBSCO)*** |  | ADD INTO SUBJCT HEADINGS:  (MH "Screen Time") OR (MH "Computers, Handheld+") OR (MH "Smartphone") OR (MH "Internet Addiction Disorder") OR (MH "Technology Addiction+") OR (MH "Internet Use") OR (MH "Television+") OR (MH "Video Games+") OR (MH "Digital Technology")  (MH "Family Relations+") OR (MH "Parent-Child Relations+") OR (MH "Maternal Behavior+") OR (MH "Paternal Behavior") OR (MH "Parents+") OR (MH "Parenting") OR (MH "Family+") OR (MH "Family Conflict") OR (MH "Family Characteristics+") OR (MH "Family Health") OR (MH "Child Rearing+") OR (MH "Mother-Child Relations+") OR (MH "Mothers+") OR (MH "Father-Child Relations") OR (MH "Fathers+") |
| ***ERIC (Via EBSCO)*** |  | ADD INTO SUBJECT HEADINGS:  DE "Outdoor Education"  DE "Handheld Devices" OR DE "Computers" OR DE "Computer Games" OR DE "Computer Use" OR DE "Internet" OR DE "Laptop Computers" OR DE "Television" OR DE "Television Viewing" OR DE "Video Games"  DE "Family (Sociological Unit)" OR DE "Parents" OR DE "Family Attitudes" DE "Caregiver Child Relationship" OR DE "Family Life" OR DE "Parent Child Relationship" OR DE "Parenting Skills" OR DE "Parenting Styles" OR “child rearing” |
| ***EMBASE*** | S1:  ((green OR nature OR forest OR outdoor OR wilderness) NEAR/4 (time OR space* OR exposure OR area* OR playground* OR environment* OR exercise* OR based OR play* OR school* OR experience* OR therap* OR bathing OR learning OR education OR immersion OR connectedness)):ti,ab  S2:  greenspace* OR 'blue space*' OR 'shinrin yoku' OR 'eco-therap*' OR greenness:ti,ab  S3:  'green space'/exp OR 'forest bathing'/exp  S4:  #1 OR #2 OR #3  S5:  ((screen OR screens OR 'electronic device*' OR computer* OR 'hand held' OR media OR tablet* OR 'mobile device*' OR 'mobile phone*' OR television* OR 'i pad*' OR ipad* OR 'touch pad*' OR touchpad* OR 'cell phone*' OR 'smart phone*' OR smartphone* OR 'i phone*' OR iphone*) NEAR/4 (use OR exposure OR time OR behavio?r* OR addict*)):ti,ab  S6:  'screen time'/de OR 'computer addiction'/exp  S7:  #5 OR #6  S8:  family OR families OR parent* OR mother* OR father* OR 'parent-child' OR carer* OR caregiver* OR guardian* OR mum* OR mom* OR dad* OR childrearing OR 'child rearing' OR maternal OR paternal:ti,ab  S9:  'child parent relation'/exp OR 'parental behavior'/exp OR 'family dynamics'/exp  S10:  #8 OR #9  S11:  #4 AND #7 AND #10 | *Do not have mapping options selected.*  ADD INTO SUBJECT HEADINGS:  'green space'/exp OR 'forest bathing'/exp  'screen time'/de OR 'computer addiction'/exp  'child parent relation'/exp OR 'parental behavior'/exp OR 'family dynamics'/exp |
| *CENTRAL (Cochrane)* | S1:  ((green OR nature OR forest OR outdoor OR wilderness) NEAR/3 (time OR space* OR exposure OR area* OR playground* OR environment* OR exercise* OR based OR play* OR school* OR experience* OR therap* OR bathing OR learning OR education OR immersion OR connectedness)):ti,ab  S2:  (greenspace* OR "blue space*" OR "shinrin yoku" OR "eco-therap*" OR greenness):ti,ab  S3:  #1 OR #2  S4:  ((screen OR screens OR 'electronic device*' OR computer* OR 'hand held' OR media OR tablet* OR 'mobile device*' OR 'mobile phone*' OR television* OR 'i pad*' OR ipad* OR 'touch pad*' OR touchpad* OR 'cell phone*' OR 'smart phone*' OR smartphone* OR 'i phone*' OR iphone*) NEAR/3 (use OR exposure OR time OR behavio?r* OR addict*)):ti,ab  S5:  Mesh Headings  S6:  #4 OR # 5  S7:  (family OR families OR parent* OR mother* OR father* OR "parent-child" OR carer* OR caregiver* OR guardian* OR mum* OR mom* OR dad* OR childrearing OR "child rearing" OR maternal OR paternal):ti,ab  S8:  Mesh Headings  S9:  #7 OR #8  S10:  #3 AND #6 AND #9 | *FOR SUBJECT HEADINGS: Use MESH terms as above for Medline.* |
